# Effects of a Community-Based, Post-Rehabilitation Exercise Program in COPD: Protocol for a Randomized Controlled Trial With Embedded Process Evaluation

**DOI:** 10.2196/resprot.5435

**Published:** 2016-05-11

**Authors:** Laura Desveaux, Marla K Beauchamp, Annemarie Lee, Noah Ivers, Roger Goldstein, Dina Brooks

**Affiliations:** ^1^ West Park Healthcare Centre Department of Respiratory Medicine Toronto, ON Canada; ^2^ Institute for Health System Solutions and Virtual Care Women's College Hospital Toronto, ON Canada; ^3^ Women's College Research Institute Toronto, ON Canada; ^4^ Rehabilitation Sciences Institute University of Toronto Toronto, ON Canada; ^5^ School of Rehabilitation Science McMaster University Hamilton, ON Canada; ^6^ Women's College Hospital Family Practice Health Centre Toronto, ON Canada; ^7^ University of Toronto Department of Family and Community Medicine Toronto, ON Canada; ^8^ University of Toronto Department of Physical Therapy Toronto, ON Canada; ^9^ University of Toronto Department of Medicine Toronto, ON Canada

**Keywords:** COPD, pulmonary rehabilitation, exercise training, maintenance, community

## Abstract

**Background:** Chronic obstructive pulmonary disease (COPD) is a leading cause of death across the world and will become increasingly common with an aging population. Pulmonary rehabilitation (PR) is an evidence-based, nonpharmacological intervention for individuals with COPD, targeting the secondary impairments of the disease. Although the benefits of participation in PR are well established, improvements in exercise tolerance and health status typically deteriorate following discharge. Challenges with long-term adherence to recommended exercise regimens are thought to explain much of this decline. Therefore, we developed a community-based exercise maintenance program for patients with COPD following discharge from PR.

**Objectives:**

This manuscript (1) outlines the intervention, (2) describes how its effectiveness is being evaluated in a pragmatic randomized controlled trial, and (3) summarizes the embedded process evaluation aiming to understand key barriers and facilitators for implementation in new environments.

**Methods:**

Participating centers refer eligible individuals with COPD following discharge from their local PR program. Consenting patients are assigned to a year-long community exercise program or usual care using block randomization and stratifying for supplemental oxygen use. Patients in the intervention arm are asked to attend an exercise session at least twice per week at their local community facility where their progress is supervised by a case manager. Each exercise session includes a component of aerobic exercise, and activities designed to optimize balance, flexibility, and strength. All study participants will have access to routine follow-up appointments with their respiratory physician, and additional health care providers as part of their usual care. Assessments will be completed at baseline (post-PR), 6, and 12 months, and include measures of functional exercise capacity, quality of life, self-efficacy, and health care usage. Intervention effectiveness will be assessed by comparing functional exercise capacity between intervention and control groups. A mixed-methods process evaluation will be conducted to better understand intervention implementation, guided by Normalization Process Theory and the Consolidated Framework for Implementation Research.

**Results:**

Based on results from our pilot work, we anticipate a maintenance of exercise capacity and improved health-related quality of life in the intervention group, compared with a decline in exercise capacity in the usual care group.

**Discussion:**

Findings from this study will improve our understanding of the effectiveness of community-based exercise programs for maintaining benefits following PR in patients with COPD and provide information on how best to implement them. If effective, the intervention represents an opportunity to transition patients from institutionally-based rehabilitative management to community-based care. The results of the process evaluation will contribute to the science of translating evidence-based programs into regular practice.

## Introduction

### Background

Chronic obstructive pulmonary disease (COPD) is a leading cause of death across the world [1] and will become increasingly common with an aging population. The prevalence of COPD increases with age and is highest in individuals aged 65 years and over [2], with a current prevalence of 20% in this age group [3]. The natural course of COPD is that of progressive worsening of airflow limitation, repeated exacerbations, respiratory failure, and premature death. Among the major chronic illnesses in Canada, COPD accounts for the highest rate of hospital admissions [4]. Coupled with the knowledge that COPD is a common and costly condition [5], long-term strategies to improve health outcomes and prevent functional decline are likely to result in a decrease in health resource usage [6].

It is well established that pulmonary rehabilitation (PR) for those who suffer from COPD results in short-term improvements in dyspnea, exercise capacity, and health-related quality of life (HRQL). However, benefits achieved through PR tend to diminish over time [7,8], often to the point that outcomes return to preintervention levels within 12 months [9,10]. Nonadherence to a maintenance home exercise program is a key factor associated with the decline in outcomes [9,11], with a 50% reduction in adherence only 9 months after completion of intensive rehabilitation, according to patient self-report [11]. While patients are encouraged to adhere to their home exercise program upon discharge, many have difficulty maintaining their exercise routine after the transition from a hospital-based rehabilitation setting to a community setting. The benefit of rehabilitation is further diminished as patients who develop acute exacerbations struggle to return to their previous level of exercise without professional guidance [11].

Consequently, there is a growing interest in developing innovative follow-up strategies to promote long-term exercise maintenance. Supervised exercise programs delivered following PR appeal to individuals with COPD [12] and are more effective than usual care for preserving exercise capacity in the medium term [13] Studies that have examined post-rehabilitation maintenance programs in patients with COPD have not shown consistent positive effect [14,15]. The optimal method for preventing functional decline following PR remains unclear, especially in individuals with moderate and severe COPD who experience frequent exacerbations.

Data from a systematic review [13] suggests that maintenance programs with higher exercise frequency and those that involved health care support, particularly after exacerbations, provided the greatest benefit. These findings complement patient-reported barriers and facilitators from qualitative work, suggesting program proximity and a scheduled, group-based format supervised by an individual who could facilitate rapid access to a health care professional were key features to promote adherence to community-based programs [16]. The potential benefits of community-based programs extend beyond the exercise component to include opportunities for social interaction among individuals who face similar challenges in their day-to-day lives and potential follow-up for those who do not adhere. An important advantage of this type of model is the transfer of wellness maintenance away from a medical environment to a fully integrated community setting.

In light of these findings, we developed a post-PR community-based exercise maintenance program for patients with COPD and completed a pilot at a single community site. Results from our pilot study demonstrated the program was feasible and well-tolerated by participants [12]. In contrast with previous studies evaluating community-based programs post-PR, our pilot work noted sustained significant improvements in physical function and HRQL at both 6 months and 1 year following PR [17]. With the success of the pilot program, a randomized controlled trial is required to determine the effectiveness of this maintenance strategy. The overall aim of this study is to evaluate the effectiveness of a post-rehabilitation community-based exercise maintenance program that uses existing community resources to provide individuals with COPD the opportunity to exercise in a community rather than an institutional setting. This research has the potential to not only prevent functional decline in those with COPD but also to improve their HRQL. Specific objectives are below:

## Objectives

1. To evaluate the effects of a 1-year community-based maintenance exercise program on functional exercise capacity in individuals with moderate to severe COPD who have completed a course of PR.
2. To determine the effects after 1 year of this intervention on secondary outcomes including HRQL, functional strength, self-reported functional status, adherence to exercise and self-efficacy.
3. To conduct a process evaluation to understand how the intervention was operationalized at each site and to identify factors that facilitated or impeded the implementation process.

## Methods

### Study Design

**Figure 1:**
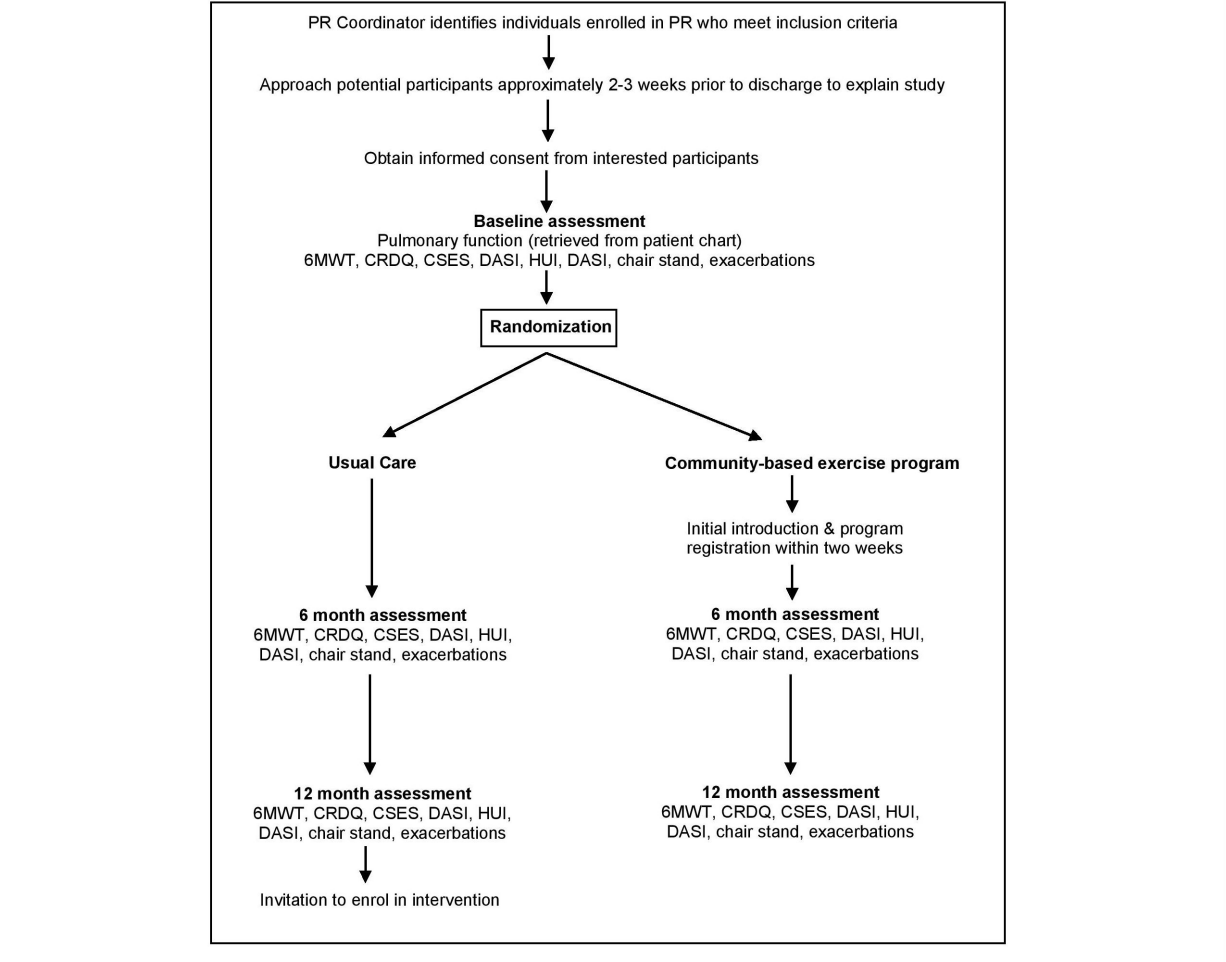
Participant flow chart (6MWT, six-minute walk test; CRDQ, chronic respiratory disease questionnaire; CSES, COPD self-efficacy scale; DASI, duke activity status index; HUI, health utility index; PR, pulmonary rehabilitation including all core components of exercise, education, self management and psychological support, in keeping with international guidelines).

The proposed study design is a two-arm, multicenter randomized controlled trial with blinding of both the outcome assessor and data analyst. A detailed participant flow chart can be found in Figure 1.

The protocol received ethics approval from the Joint Bridgepoint/West Park/Toronto Central Community Care Access Centre Research Ethics Board (REB); the Trillium Health Partners REB; the Lakeridge Health REB; the University Health Network REB; and the St. Joseph’s Care Group REB. The trial is registered with ClinicalTrials [NLM Identifier: NCT01942499].

### Setting

In the province of Ontario, many health care services are covered under the Ontario Health Insurance Program (OHIP), a government-run health plan that is publicly funded. OHIP covers medically necessary services provided by physicians, including basic and emergency services, specialist visits, and formal rehabilitation (which includes PR). Community-based exercise programs are not covered under OHIP, and can be found in both municipal and private facilities.

### Participants

Individuals with COPD will be considered eligible for the study if they: (1) have moderate to severe COPD based on international (GOLD) criteria [18], (2) are clinically stable as determined by their respirologist, (3) have completed PR within the previous 2 weeks, and (4) are able to provide their own informed consent. Participants will be given written study information for review and will completed a written consent form prior to study enrollment. All participants will be under the active care of a respirologist.

Individuals will be excluded if they have associated medical conditions that significantly limit their ability to exercise. Specifically, participants who report a history of significant cardiovascular disease (ie, congestive cardiac failure, history of cardiac arrest, acute myocardial infarction within the preceding 3 months, symptomatic ischaemic cardiac disease, or uncontrolled systemic hypertension) or report severe nonrespiratory symptoms during exercise will be excluded from the study. Participants receiving mechanical ventilation and those who are unable or unwilling to attend the community-based exercise program or follow-up assessment sessions will also be excluded.

### Allocation

A computer-generated randomization schedule using variable block size will be created. A member of the research team will consecutively randomize participants using sealed, opaque envelopes. Randomization will be stratified by the use of supplemental oxygen to minimize the impact of this factor, as it has been shown to influence the response to PR [19] and improve the total distance achieved during the 6-minute walk test (6MWT) by 12 to 59 m [20,21].

### Intervention

The course of PR completed by both groups is in accordance with international guidelines [8] and will consist of all core components of rehabilitation including exercise, education, self-management, and nutrition, as well as psychological support. Thus, all participants will possess the tools and knowledge necessary to manage their condition prior to enrolment in the community maintenance program. Post-PR, participants randomized to the intervention group will receive the community-based maintenance exercise program for 12 months.

#### Control: Usual Care

Patients in both groups will receive usual care by their family physician and respiratory specialist (ie, medical care or prescriptions will not be standardized). Post-PR, they will receive the standard home exercise instructions and regular appointments with a physical therapist to review their program and address any outstanding issues.

#### Intervention: Community-Based Exercise Program

The intervention group will receive the same usual care and follow-up as the control group. In addition, they will be enrolled in a community-based maintenance exercise program for 12 months. Participants will be asked to attend a minimum of two sessions per week and are able to attend more frequently if they so choose. Each session will be approximately 60 minutes in duration. The exercise program will be delivered at one of seven community centers affiliated with the study; location selection will be based on the patient’s preference and proximity to their home. Each participant will receive a full, 1-year membership to the community center as part of the study.

Group-based classes led by a certified fitness instructor will be offered at least twice per week, although participants will have the option of attending the community center at an alternative time if they are unable to attend the group class. Participants who attend the center outside of class times will have access to a certified fitness instructor for supervision. Fitness instructors will be certified by the Canadian Society for Exercise Physiology, the principal body for physical activity, health and fitness research, and personal training in Canada. Educational materials and training workshops regarding issues pertinent to supervising patients with COPD during exercise, symptom management, and guidelines for training progression will be provided to fitness instructors prior to launching the study.

The content of each exercise program will be individualized according to each participant’s specific needs. Each exercise session will include a component of aerobic exercise such as walking or cycling, upper-limb resistance exercise (eg, free weights for bicep curls and triceps extensions), and activities designed to optimize balance, flexibility and strength (eg, functional exercises such as minisquats, stairs, basic stretches, core strengthening) based on PR guidelines (see Table 1 for an example). In addition to attending the twice weekly exercise program, participants will be encouraged to continue with their home exercise program consisting of aerobic exercise and strengthening. As we expect an inherent degree of variability in program delivery across sites, individual program details will be evaluated and reported as part of the process evaluation in order to capture variation across sites.

**Table 1:** Example of exercise program at community center.

Type	Approximate duration	Potential activities
Warm up	10 minutes	Gentle stretches for all major muscle groups (neck, shoulders, arms, hamstrings, quadriceps, and calves); marching on the spot to increase heart rate.
Aerobic training	20-30 minutes	Walking along a designated track with rests as needed, cycling and/or treadmill.
Functional exercises to promote strength and balance	20-30 minutes	Free weights and ‘wall climbing’ for upper extremity; mini-squats, stairs, hip abduction and hip extension while holding onto the back of a chair (therabands available to add resistance) for lower extremity; basic balance exercises such as practicing tandem stance, standing on one leg, walking on different surfaces (with mats and rails available for safety).
Cool down	10 minutes	Gentle stretches for all major muscle groups (neck, shoulders, arms, hams, quads, and calves); slow walking to decrease heart rate.

The case manager, a member of the research team and a clinically trained physiotherapist, will have training in pulmonary rehabilitation and be familiar with the rehabilitation programs offered at each site. The case manager will collaborate with the patient’s PR physiotherapist and community center fitness instructor to establish the initial frequency, intensity, and training modalities for the community exercise program, tailored to the individual’s capacity. During the 12-month community-based program, the case manager will remain in communication with participants and fitness staff via a study phone-line and email. Participants attending exercise classes will be asked to contact the case manager by telephone after an absence of more than 1 week, due to illness or other reasons, so that support may be provided in managing exacerbations and resuming regular exercise. If significant new health problems arise, participants will be encouraged to return to their family doctor or respiratory specialist for review. Preliminary results from our pilot study demonstrated that the case manager was only accessed on occasions where a patient required additional support in managing exacerbations and where the medical safety of the patient was unclear to the fitness instructor. Feedback from focus groups with participants indicated that the presence of a case manager was a valued component of the model [12]. The role of the case manager moves this model into an integrated care strategy by providing patients and fitness staff with access to a health care professional when needed.

## Outcomes

### Primary Outcome

#### Six-Minute Walk Test

The 6MWT is a valid, responsive, interpretable, self-paced test that quantifies functional exercise capacity in terms of the distance walked in 6 minutes (6-minute walk distance) in patients with COPD [22]. The test will be performed over a 30-m level, straight course within an enclosed corridor, using the protocol described by the American Thoracic Society [22]. Outcome assessors will receive standardized training and will be blinded to group allocation. The measurement properties of this test have been well established in the COPD population [23]. A minimum important difference (MID) for the 6MWT is 54 m [24].

### Secondary Outcomes

#### Chronic Respiratory Disease Questionnaire

Health-related quality of life will be measured using the Chronic Reparatory Disease Questionnaire (CRQ). The CRQ is a disease-specific instrument evaluating four domains that are considered important to individuals with chronic airflow limitation [25,26]. Participants will be required to quantify events and experiences that have taken place over the 2-week period preceding administration of the questionnaire. It includes 20 questions in four domains: dyspnea, fatigue, emotional function, and mastery. Answers are scored on a 7-point scale ranging from 1 (maximum impairment) to 7 (no impairment). The results are expressed as the mean score for each domain and the mean overall score. The MID for the CRQ is a change (improvement or deterioration) of 0.5 per item [27]. The CRQ is valid, responsive and interpretable when used among patients with COPD [25-27].

#### Duke Activity Status Index

The Duke Activity Status Index (DASI) is a 12-item questionnaire that requires only simple yes/no responses and takes less than 5 minutes to complete [28]. The questionnaire includes activities representative of personal care, ambulation, household tasks, sexual function, and recreational activities. The DASI has high criterion validity for predicting functional outcomes in patients with moderate to severe COPD [29] and was responsive to change in our pilot study [17].

#### Lower Extremity Functional Strength

The repeated chair stand test (number of sit-to-stands the subject can complete in a 30-second time-period) will be used as a measure of functional lower body strength. Reliability and validity of this measure has been previously evaluated in community-dwelling older adults [30] and in people with COPD [31] and it has been shown to be correlated to maximal voluntary force from a seated leg press [30,31].

#### Self-Efficacy

Behavioural modification is embedded in the rehabilitation process. Bandura describes the concept of self-efficacy as the ‘belief in one’s capabilities to organise and execute the course of action required to produce given attainments’ [32]. A specific self-efficacy scale has been designed and validated for COPD, the COPD self-efficacy scale (CSES) [33], and was recently shown to be responsive to the effects of PR [34]. It is a 34-item questionnaire divided into five sections, one of which pertains to exercise.

#### Exacerbations

Acute exacerbations will be defined based on symptoms according to the criteria described by Anthonisen and colleagues [35], which are increased dyspnea with changes in sputum purulence or volume lasting at least 2 consecutive days. We will use intervention-based criteria for classifying the exacerbation as mild, moderate, or severe, depending on whether they are managed at home with no additional health care contact (mild), at home with unscheduled health care contact or the initiation of oral corticosteroids or antibiotics (moderate), or in the emergency room or hospital (severe) [35]. Patients will be asked to self-report this information at each assessment.

### Data Analysis

Descriptive summary statistics will be reported using means and standard deviations, with median values as indicated for nonparametric data. The primary analysis will use a generalized linear model to examine the effect of treatment, time (follow-up at 6 and 12 months), and the interaction between treatment group and time. Values at baseline will be used as a covariate in this analysis. Secondary analyses will include adjustment for other baseline variables including age, sex, forced expiratory volume_**1**_, and exacerbations. The generalized linear model creates a working covariance matrix for the model parameters that deals with missing data, including patients who do not complete all follow-up measures. The model uses all data in calculating a net effect of intervention versus control at 12 months and an estimate of precision (95% confidence interval) at that final follow-up. This analytic strategy will be used for all continuous variables that are primary or secondary measures of outcome (ie, 6MWD, CRQ domains, CSES, DASI, chair stand test). Data will be analyzed using the Statistical Package for the Social Sciences, version 22.0, with significance set at *P*<0.05.

#### Sample Size Calculation

Sample size calculations are based on the primary outcome measure, the 6MWT. We calculated sample size using a paired samples t-test. As the repeated measures analysis of the variance will be a more powerful analysis than a t-test, our sample size is a conservative estimate. Furthermore, as we will use baseline scores as a covariate in our planned analysis, we will be using within participant variability as our error (and not within participant variability), making our estimate even more conservative. A sample size estimation based on the knowledge of the differences in 6MWD that represent a clinically important difference (54 m and standard deviation of 86 m [15]), type I error of 0.05 and a power of 80%, revealed the need for a sample size of 40 in each group for a total of 80 participants. From our previous experience in this population, we estimate the rates of noncompliance and loss to follow-up to be 15% to 20% (8-10 participants per group). Therefore, we will aim to recruit 100 participants.

### Process Evaluation

The process evaluation was informed by the MRC Guidance on Process Evaluations of complex interventions [36] and will focus on the evaluation of fidelity and implementation context. Intervention fidelity will be monitored throughout the study through semiannual check-ins with community facilities. Facilities will explicitly outline the operationalization of the intervention at their respective facility, including frequency, duration, supervision, attendance monitoring, and individual program components. Implementation will be explored through the integration of two frameworks: (1) Normalization Process Theory (NPT) [37] will be used to understand how the intervention was operationalized and (2) the Consolidated Framework for Implementation Research (CFIR) [38] will be used to evaluate contextual factors that influence the adoption, implementation, and maintenance of the intervention.

NPT is an established framework for understanding how and whether complex interventions become embedded in routine practice (ie, normalized) [37].This approach is ideally suited to this study as it entails numerous individuals, professionals, and organizations that may impact the effectiveness of a community-based exercise program. A quantitative questionnaire [39] will be administered following completion of the study. The questionnaire will evaluate the extent to which individuals involved in delivering the intervention (ie, PR and community center staff) make sense of the work of implementing and integrating the intervention (coherence); how they engage with it (cognitive participation); how they enact it (collective action); and how they appraise its effects (reflexive monitoring).

The CFIR will be used to develop a semistructured interview guide; PR coordinators and staff involved in referring participants, as well as managers, and fitness instructors from each community site will be invited to participate. Interviews will be conducted during the post-implementation phase and will elicit information relating to the experience of implementing and administering the intervention. Perspectives around program sustainability in order to inform broader implementation, should the program be effective, will also be explored.

For quantitative data (eg, survey results) descriptive analyses, including frequencies, means, and percentages will be performed using the Statistical Package for the Social Sciences version 22.0. Summary indices will be calculated for each NPT construct in order to evaluate the degree to which the intervention has become part of routine practice. Qualitative data collected during interviews will be audio recorded and transcribed verbatim. After reading the transcripts several times to become familiar with the text, codes will be identified and subsequently categorized into different themes [40]. Analysis will involve mapping the themes to the CFIR Framework to identify points of convergence (pattern matching) and divergence (examining alternative explanations). Sampling will continue until saturation is reached. Qualitative analysis will be performed using NVivo software.

### Results

The trial is currently recruiting participants and will continue until the proposed sample size is reached. Approximately one-half of the required participants are enrolled in the study to date, indicating that data collection is likely to continue until 2018. Fidelity to the implementation process across all five hospitals and seven community sites is being monitored throughout the study. The rest of the process evaluation will commence in 2016.

## Discussion

### Trial Implications

This study will determine the effectiveness of a community-based maintenance exercise intervention for preserving functional exercise capacity following PR in patients with moderate to severe COPD. Upon completion, this study will be the first multicenter, randomized controlled trial of community-based maintenance programs following PR. Furthermore, this is the first study of maintenance exercise in COPD that includes a formal process evaluation, which will identify context-specific factors related to program implementation, thereby facilitating the uptake of the program into new environments. The intervention is fairly simple to implement, can be delivered in existing community settings, and requires minimal health system support. Although several studies have reported limited success with maintenance exercise programs following PR [11,14,15,41], the intervention outlined in this study is positioned for improved outcomes as we have incorporated previously identified elements of successful maintenance exercise programs and are building on an effective model of maintenance from our pilot study [17]. Specifically, our delivery model includes a higher exercise frequency [13], supervised exercise [12,16], program proximity [12,16], and health care professional support [12,13,16].

If participation in a community-based maintenance exercise program results in improved maintenance of functional exercise capacity compared with standard care, this approach will represent an innovative (and relatively inexpensive) strategy to optimize the maintenance of gains made during PR. Our project is particularly relevant for guiding both clinical and policy-based decision-making, given the large population of adults with moderate and severe COPD who cannot be served on an ongoing basis by existing PR programs. Furthermore, a community-based approach using case managers could offer a scalable approach for maintaining well-being across multiple disease-conditions post-rehabilitation.

### Conclusions

In conclusion, this study will provide definitive evidence on the effectiveness of a community-based maintenance exercise program for patients with COPD. The intervention involved in the current study was designed to address factors that contribute to success previously identified in the literature within the constraints of existing community resources to maximize generalizability. The addition of a process evaluation is an added strength that will offer insight into the factors that may impede or facilitate the implementation of the program. Data from this evaluation will provide information around the potential application of the intervention across different health care and community settings.

## Appendix

Multimedia Appendix 1 CIHR Operating Grant Reviews.

## Abbreviations

6MWT: 6-minute walk test
CFIR: consolidated framework for implementation research
COPD: chronic obstructive pulmonary disease
CRQ: chronic respiratory disease questionnaire
CSES: COPD self-efficacy scale
DASI: duke activity status index
HRQL: health-related quality of life
MID: minimum important difference
NPT: normalization process theory
OHIP: Ontario Health Insurance Program
PR: pulmonary rehabilitation
REB: research ethics board
