# Supplementary material for: Effects of a Community-Based, Post-Rehabilitation Exercise Program in COPD: Protocol for a Randomized Controlled Trial With Embedded Process Evaluation
Source: JMIR Res Protoc. 2016 May 11;5(2):e63. doi: 10.2196/resprot.5435 (PMC4880740; doi:10.2196/resprot.5435)
Supplement: Multimedia Appendix 1 [file resprot_v5i2e63_app1.pdf]

Canadian Institutes of Health Research/Instituts de recherche en santé du Canada

Notice of Recommendation/Avis de recommandation

Application Number/Numéro de la demande: 300025

Committee Code/Code du comité: RS

Applicants/Candidats: Dr. Dina BROOKS Dr. Roger S. GOLDSTEIN

With/Avec: Dr. M. BEAUCHAMP Ms. L. DESVEAUX Dr. G. GUYATT

Institution paid/ Établissement payé: University of Toronto

Title/Titre: Randomized controlled trial of a community-based exercise program for preventing functional decline among individuals with chronic obstructive pulmonary disease

Primary Inst./Inst. principal: Circulatory and Respiratory Health

Other Related Inst./ Autres inst. connexes: Aging

Competition /Concours: Operating Grant
March/Mars 01, 2013

Number in competition/Nbre de demandes dans le concours: 2253

Peer Review Committee Recommendation, for your information and use/
Recommandation du comité d'examen par les pairs, pour fins d'information et d'utilisation:

**Committee/Comité:**	Respiratory System
**Number reviewed/ Demandes examinées:**	47
**Application rank within the committee/ Rang de la demande dans le comité:**	2
**Percent Rank within the committee / Rang en pourcentage au sein du comité:**	4.26%
**Rated / Cote:**	4.68
**Recommended Term/ Durée recommandée:**	3 years/ans 0 months/mois
**Recommended average annual operating amount/ Montant annuel moyen recommandé pour le fonctionnement:**	$94,688
**Recommended equipment amount/ Montant recommandé pour les appareils:**	$0

This document is for information only.
An application rated below 3.50 is ineligible for CIHR funding. For applications rated 3.50 and above, please note that it is the application's rank within the peer review committee that determines whether it is funded, rather than its absolute rating. The final funding decision will be communicated in the Notice of Decision.

Document à titre d'information seulement.
Une demande cotée en dessous de 3,5 n'est pas admissible au financement des IRSC. En ce qui a trait aux demandes cotées 3,50 ou plus, veuillez noter que l'on détermine l'attribution des fonds en fonction du classement obtenu au sein du comité d'examen par les pairs plutôt qu'en fonction du classement absolu. La décision finale relative au financement sera communiquée dans l'Avis de décision.

Canadian Institutes of Health Research / Instituts de recherche en santé du Canada

Notice of Decision / Avis de décision

Application Number/Numéro de la demande: 300025
Committee Code/Code du comité: RS

Applicants/Candidats: Dr. Dina BROOKS   Dr. Roger S. GOLDSTEIN

With/Avec: Dr. M. BEAUCHAMP   Ms. L. DESVEAUX   Dr. G. GUYATT

Institution paid/ Établissement payé: University of Toronto

Title/Titre: Randomized controlled trial of a community-based exercise program for preventing functional decline among individuals with chronic obstructive pulmonary disease

Primary Inst./ Inst. principal: Circulatory and Respiratory Health

Other Related Inst./ Autres inst. connexes: Aging

**Competition Outcome/Résultats du concours:**	Operating Grant<br>March/Mars 01, 2013
**Number in competition/Nbre de demandes dans le concours:**	2253
**Number approved/Nbre de demandes approuvées:**	400

**Decision on your application/ Décision sur votre demande:**	Approved
**Average annual amount/ Montant annuel moyen:**	$71,489
**Equipment amount/ Montant pour les appareils:**	$0
**Term/Durée:**	**3 yrs/ans 0 months/mois**

Peer Review Committee Recommendation, for your information and use/ Recommandation du comité d'examen par les pairs, pour fins d'information et d'utilisation:

**Committee/Comité:**	Respiratory System
**Number reviewed/ Nbre de demandes examinées:**	47
**Number approved in that committee/ Nbre de demandes approuvées dans ce comité:**	8
**Application rank within the committee/ Rang de la demande dans ce comité:**	2
**Percent Rank Within the Committee/ Rang en pourcentage au sein du comité:**	4.26%
**Rating/ Cote:**	4.68
**Recommended average annual amount/ Montant annuel moyen recommandé:**	$94,688
**Recommended equipment amount/ Montant recommandé pour les appareils:**	$0

*** Applications receiving a score of less than 3.5 on any evaluation criteria will not be considered for Funding. / Les demandes qui ont reçu une note inférieure à 3.5 pour n'importe quel des critères d'évaluation ne sont pas admissibles.

This document is for information only. Official payment is stated on the CIHR Authorization for Funding.
Document à titre d'information seulement. Le paiement officiel est indiqué sur l'autorisation de financement des IRSC.

CIHR IRSC

Institute of Aboriginal Peoples' Health
Institute of Aging
Institute of Cancer Research
Institute of Circulatory and Respiratory Health
Institute of Gender and Health
Institute of Genetics
Institute of Health Services and Policy Research
Institute of Human Development and Child and Youth Health
Institute of Infection and Immunity
Institute of Musculoskeletal Health and Arthritis
Institute of Neurosciences, Mental Health and Addiction
Institute of Nutrition, Metabolism and Diabetes
Institute of Population and Public Health

Institut de la santé des Autochtones
Institut du vieillissement
Institut du cancer
Institut de la santé circulatoire et respiratoire
Institut de la santé des femmes et des hommes
Institut de génétique
Institut des services et des politiques de la santé
Institut du développement et de la santé des enfants et des adolescents
Institut des maladies infectieuses et immunitaires
Institut de l'appareil locomoteur et de l'arthrite
Institut des neurosciences, de la santé mentale et des toxicomanies
Institut de la nutrition, du métabolisme et du diabète
Institut de la santé publique et des populations

June 28, 2013

Dr. Dina BROOKS
Department of Physical Therapy
160-500 University Avenue
Toronto, Ontario M5G 1V7

Dear Dr. BROOKS:

We are pleased to inform you that the Canadian Institutes of Health Research (CIHR) has approved your recent application entitled "Randomized controlled trial of a community-based exercise program for preventing functional decline among individuals with chronic obstructive pulmonary disease". If you are receiving this letter through ResearchNet, your Authorization for Funding will follow in the mail otherwise it is enclosed in this package.

If you have not already received the review documents related to your proposal, please contact us. Should you have any questions about the review process, please address them directly to CIHR staff. Do not contact the officers or members of the peer review committee. As CIHR does not notify co-applicants of the decision, we ask that you inform those individuals involved, along with their research institutions (if different from your own), of the outcome of this application.

Congratulations on your success in this competition.

Martine Lafrance

Martine Lafrance, Ph.D.
Deputy Director, Open Programs
Research and Knowledge Translation Portfolio

331736-201303MOP-RS-300025-35000-DLGAR

Institute of Aboriginal Peoples' Health

Institute of Aging

Institute of Cancer Research

Institute of Circulatory and Respiratory Health

Institute of Gender and Health

Institute of Genetics

Institute of Health Services and Policy Research

Institute of Human Development and Child and Youth Health

Institute of Infection and Immunity

Institute of Musculoskeletal Health and Arthritis

Institute of Neurosciences, Mental Health and Addiction

Institute of Nutrition, Metabolism and Diabetes

Institute of Population and Public Health

Institut de la santé des Autochtones

Institut du vieillissement

Institut du cancer

Institut de la santé circulatoire et respiratoire

Institut de la santé des femmes et des hommes

Institut de génétique

Institut des services et des politiques de la santé

Institut du développement et de la santé des enfants et des adolescents

Institut des maladies infectieuses et immunitaires

Institut de l'appareil locomoteur et de l'arthrite

Institut des neurosciences, de la santé mentale et des toxicomanies

Institut de la nutrition, du métabolisme et du diabète

Institut de la santé publique et des populations

June 28, 2013

Dr. Dina BROOKS
Department of Physical Therapy
160-500 University Avenue
Toronto, Ontario M5G 1V7

Dear Dr. BROOKS:

Congratulations on your success in the recent Canadian Institutes of Health Research funding competition.

You should take great pride in your success, particularly in light of the very competitive nature of CIHR peer review.

As you know, peer review is the cornerstone of our research funding system. This process rests on the kind voluntarism of your colleagues at other institutions who generously gave their time to review your application.

The Canadian Institutes of Health Research is committed to building an innovative national health research enterprise. To this end we have initiated a process to design a new Open Suite of Programs and peer review system to ensure the long-term sustainability of CIHR's contribution to the Canadian health research enterprise, to remove barriers, and to enable researchers from all pillars to improve CIHR's ability to deliver on its mandate.

To meet CIHR goals, we must also continue to communicate the value of health research to Canadians. That is why we encourage you to work with your institution to promote your research . We have developed guidelines on public communication, available on our website at http://www.cihr-irsc.gc.ca/e/30789.html, to support you in this activity.

Once again, congratulations and I wish you success in your research.

Yours sincerely,

Alain Beaudet, MD, Ph.D.
President

331740-201303MOP-RS-300025-35000-CONGR

President

Canadian Institutes of Health Research
Room 97, 160 Elgin Steet, Address locator: 4809A
Ottawa, (Ontario) K1A 0W9 Tel.: (613) 941-2672
Fax (613) 954-1800 **www.cihr-irsc.gc.ca**

Président

Instituts de recherche en santé du Canada
Pièce 97, 160 rue Elgin, Indice de l'adresse: 4809A
Ottawa, (Ontario) K1A 0W9 Tél.: (613) 941-2672
Fax (613) 954-1800 **www.irsc-cihr.gc.ca**

Canada

Review Type/Type d'évaluation: Committee Member 1/Membre de comité 1
Name of Applicant/Nom du chercheur: BROOKS, Dina
Application No./Numéro de demande: 300025
Agency/Agence: CIHR/IRSC
Competition/Concours: 2013-03-01 Operating Grant/Subvention de fonctionnement
Committee/Comité: Respiratory System/Appareil respiratoire
Title/Titre: Randomized controlled trial of a community-based exercise program for preventing functional decline among individuals with chronic obstructive pulmonary disease

Assessment/Évaluation:

PI: Dina Brooks

Review of the Protocol:

This is a protocol for a randomized, controlled trial of a community-based exercise program in patients with COPD. The investigators will randomize 100 subjects with COPD who have completed the pulmonary rehab programme at Westpark Hospital in Toronto. Subjects will be randomized to a community-based exercise program to be done in one of 3 community centers in Toronto at least twice per week for one year.

The primary objectives are to evaluate the effects over one year of a community-based maintenance exercise program on functional exercise capacity as measured by six-minute walk distance (in individuals with moderate to severe COPD who have completed a course of pulmonary rehabilitation).

The program will be supervised by fitness instructors, and a case manager will provide remote supervision. This will be a single-blind trial, and there will be concealment of randomization and the outcome assessor will remain unaware of the patient group allocations. The primary outcome will be the 6 minute walk test results. Quality of life as assessed by the Chronic Respiratory Disease Questionnaire will be a secondary outcome, a cost-effectiveness assessment is also built into the analysis protocol.

Review of the team:

The principal applicant is a PhD researcher who is a Professor at University of Toronto Dept of Physical Therapy. Dr. Brooks is an accomplished researcher with an interest in exercise and rehabilitation of patients with chronic lung disease. She has over 80 publications in the past 5 years, many in high-impact journals. She supervises numerous graduate students. The PI is an experienced accomplished investigator who is clearly capable of running this study. Her co-applicant Dr. Roger Goldstein is a senior clinical researcher with expertise in pulmonary rehabilitation. Dr. Goldstein will be able to provide adequate access to patients, and will provide mentorship for the study team. Dr. Gordon Guyatt is on the team and he will provide

Review Type/Type d'évaluation: Committee Member 1/Membre de comité 1
Name of Applicant/Nom du chercheur: BROOKS, Dina
Application No./Numéro de demande: 300025
Agency/Agence: CIHR/IRSC
Competition/Concours: 2013-03-01 Operating Grant/Subvention de fonctionnement
Committee/Comité: Respiratory System/Appareil respiratoire
Title/Titre: Randomized controlled trial of a community-based exercise program for preventing functional decline among individuals with chronic obstructive pulmonary disease

---

Assessment/Évaluation:

biostatistical and methodologic support. The team is strong and able to carry out the proposed study.

Strengths of the Application:

1) The researchers are studying an important clinical problem. The applicants have convincingly shown that although individuals with COPD demonstrate marked improvements following participation in a formal rehabilitation program, they often experience a decline in function over the subsequent months. Interventions would be required to keep these patients functioning at a high level.

2) The team is very strong and capable of completing this study.

3) The study will include a formal economic analysis of the exercise intervention strategy. The measure of effectiveness for the economic analysis will be quality-adjusted life-years (QALYs) and the relevant utility weights will be obtained from the patients. The two groups will be compared in terms of the mean utility-weighted follow-up time, the difference being the gain in expected QALYs per patient associated with the maintenance intervention.

4) The applicants have results from a previous study they conducted which showed that enhanced follow-up (without a specific exercise program) did not improve outcomes in patients who had completed a standard pulmonary rehab program, because of non-adherence to home exercise. Their previous study provides rationale and justification for the current application.

5) The applicants have developed a post-rehabilitation community-based exercise program in partnership with the City of Toronto, Parks, Forestry and Recreation Division and piloted the program at a single community site. They have preliminary results which demonstrate the feasibility of the program in 29 patients (who have all been treated with the intervention), with sustained significant improvements in 6MW distance and HRQL 6-months and 1-year following PR. They have provided the pilot study results in detail in section 2.19 of the revised grant.

6) The applicants have responded to the previous two courses of reviews and they have appropriately addressed previous methodologic limitations. Specifically they have justified their between group difference in 6 MW test (the primary outcome) of 54 m. From their pilot data it appears that at 12 months subjects improved their 6MW distance by approximately 35 meters. They expect subjects in the control group to decline by as much as 60 meters over 12 months. Therefore the between group difference of 54 m (the

Review Type/Type d'évaluation: Committee Member 1/Membre de comité 1
Name of Applicant/Nom du chercheur: BROOKS, Dina
Application No./Numéro de demande: 300025
Agency/Agence: CIHR/IRSC
Competition/Concours: 2013-03-01 Operating Grant/Subvention de fonctionnement
Committee/Comité: Respiratory System/Appareil respiratoire
Title/Titre: Randomized controlled trial of a community-based exercise program for preventing functional decline among individuals with chronic obstructive pulmonary disease

---

Assessment/Évaluation:

MCID) should be achievable with the intervention, and the sample size is appropriate.

Weaknesses of this application : None are apparent.

Overall assessment:

This is a strong grant, by a strong team. They are studying an important problem; it is well known that benefits of pulmonary rehab are not sustained once patients exit their program, because of non-adherence to home exercise. The intervention described in this protocol, a community-based exercise program, has the potential to extend the long-term benefits of exercise/rehab to the COPD population. The authors have presented pilot data that shows that they can deliver the intervention, and this pilot data suggests feasibility of the study. The authors have improved their protocol significantly compared to the previous 2 submissions. I am in favour of funding the current protocol.

Review Type/Type d'évaluation: Committee Member 2/Membre de comité 2
Name of Applicant/Nom du chercheur: BROOKS, Dina
Application No./Numéro de demande: 300025
Agency/Agence: CIHR/IRSC
Competition/Concours: 2013-03-01 Operating Grant/Subvention de fonctionnement
Committee/Comité: Respiratory System/Appareil respiratoire
Title/Titre: Randomized controlled trial of a community-based exercise program for preventing functional decline among individuals with chronic obstructive pulmonary disease

---

Assessment/Évaluation:

Synopsis of the Proposal

This is a resubmission of a proposal previously reviewed by the Respiratory Systems Committee and received a very high score.

This novel and very clinically relevant proposal addresses the effect of a community based exercise program on the exercise tolerance and health-related quality of life in individuals with COPD. The applicants propose to randomize subjects in a ”blinded” manner to either standard care, or a 12 month exercise program supervised by a qualified fitness instructor employed by the Toronto Parks and Recreation Department, at one of 3 local community centers. The subjects will be evaluated at 0, 6 and 12 months with a 6 minute-walk-test. Secondary measures to be evaluated include the chronic respiratory disease questionnaire, an activity status index, sit- to-stand test and health-care utilization rates.

The investigator team is highly qualified and multi-disciplinary in nature. The two principal investigators have a complementary background, the first being physical therapist and the second a respiratory medicine specialist.

Assessment of the Proposal

This highly focused proposal addresses a novel and promising intervention that if proven successful could significantly impact on the quality of life of subjects with COPD and their disease-associated health-care costs.

The rationale for the study stems from the fact that individuals with COPD while hospitalized follow a proper physical therapy program, yet the compliance rate with home-exercise is poor. Such lack of at home exercise compliance results in limited exercise tolerance and negatively impacts on quality of life measures.

Other studies have shown evidence of a positive impact of a structured exercise program for post-hospital discharge COPD subjects. Yet, the available data refers to exercise program ran by medical personnel and thus financially costly. The applicants propose to develop a community based, fitness instructor-managed exercise program for individuals with COPD. 280 subjects will be recruited over a 24 month period and randomized to either a standard treatment that reflects present management of subjects with COPD, or the intervention treatment. The subjects in the experimental group will be required to attend at least twice a week, a community based qualified fitness instructor program to increase exercise tolerance to a number of well

Review Type/Type d'évaluation: Committee Member 2/Membre de comité 2
Name of Applicant/Nom du chercheur: BROOKS, Dina
Application No./Numéro de demande: 300025
Agency/Agence: CIHR/IRSC
Competition/Concours: 2013-03-01 Operating Grant/Subvention de fonctionnement
Committee/Comité: Respiratory System/Appareil respiratoire
Title/Titre: Randomized controlled trial of a community-based exercise program for preventing functional decline among individuals with chronic obstructive pulmonary disease

Assessment/Évaluation:

established and proven exercise routines. An assessor blinded to the group assessment will evaluate these subjects at timed intervals. The primary and secondary outcome measures are clearly defined, as well as inclusion and exclusion criteria. The applicants have elegantly designed the trial and ensured that the subject assessment bias will be minimized, or prevented altogether. The power analysis appears to ensure that an adequate sample size will be recruited to adequately evaluate the primary outcome measures.

Relatively minor concerns were raised by the reviewers during the previous submission. The applicants have, in the opinion of this reviewer, adequately addressed all these concerns. Preliminary data obtained by the investigators show sufficient evidence that the hypothesis is sound.

Interestingly and to great credit to the applicants, a detailed economic analysis of the impact of such exercise program on health care utilization rates, as well as quality of life of the subjects will be conducted. It is estimated that such exercise program, if indeed proven to be efficacious would result in a positive impact on both quality of life of COPD subjects and their health care costs.

The investigator team is highly qualified and their complementary expertise is indeed a great asset to this study. No significant weaknesses were identified with the present proposal.

Budget

The applicants request a full salary for research assistant, a technician and graduate student. The personnel needs are well justified and appropriate. The expendables and service items are equally justified and relatively small.

Review Type/Type d'évaluation: SO Notes /Notes de l'agent scientifique
Name of Applicant/Nom du chercheur: BROOKS, Dina
Application No./Numéro de demande: 300025
Agency/Agence: CIHR/IRSC
Competition/Concours: 2013-03-01 Operating Grant/Subvention de fonctionnement
Committee/Comité: Respiratory System/Appareil respiratoire
Title/Titre: Randomized controlled trial of a community-based exercise program for preventing functional decline among individuals with chronic obstructive pulmonary disease

---

Assessment/Évaluation:

Strengths: The proposal is well crafted. The study goals are clearly laid out and methodologically sound. Clear strengths are the principle applicant and her team of collaborators. The research question is topical and addresses an important clinical need. Concerns raised in previous reviews were carefully addressed.

Weaknesses: There were no significant weaknesses discussed.

Budget was very reasonable.
